# Generational Differences in Age-Specific Dementia Prevalence Rates

**DOI:** 10.1001/jamanetworkopen.2025.13384

**Published:** 2025-06-02

**Authors:** Xiaoxue Dou, Sabrina Lenzen, Luke B. Connelly, Ruozhou Lin

**Affiliations:** 1 Centre for the Business and Economics of Health, The University of Queensland, Brisbane, Queensland, Australia; 2 School of Economics, The University of Queensland, Brisbane, Queensland, Australia; 3 Department of Sociology and Business Law, The University of Bologna, Bologna, Italy; 4 School of Energy and Environment, City University of Hong Kong, Hong Kong

## Abstract

**Importance:**

Dementia is a significant public health challenge, with its prevalence expected to increase as life expectancy continues to extend globally.

**Objective:**

To estimate generational differences in age-specific dementia prevalence across the US, Europe, and England.

**Design, Setting, and Participants:**

This cross-sectional study used data from 1994 to 2021 from the United States Health and Retirement Study (HRS), from 2004 to 2020 from the Survey of Health, Ageing and Retirement in Europe (SHARE), and from 2002 to 2019 from the English Longitudinal Study of Ageing (ELSA). Statistical analysis was performed from May 2023 to February 2025.

**Main Outcomes and Measures:**

First, 2 different methods—the validated algorithm and machine learning techniques as the robustness check—were used to identify respondents with probable dementia. Then the status of dementia was used to estimate the association of cohort with dementia status by age-period-cohort methodology.

**Results:**

Of the 99 420 individuals (mean [SD] age, 79.1 [6.2] years; 59.2% women) in the HRS dataset, 21 069 (21.2%) were included in this study; of the 83 580 individuals (mean [SD] age, 78.6 [5.9] years; 54.7% women) in the SHARE dataset, 32 490 (38.9%) were included in this study; and of the 31 384 individuals (mean [SD] age, 77.9 [5.6] years; 55.8% women) in the ELSA dataset, 8878 (28.3%) were included in this study. Focusing on people aged 71 years or older and controlling for age and period effects, birth cohorts born more recently were less likely to develop dementia in all 3 regions, albeit at different rates (point estimate for 1944-1948 vs 1919-1923 cohorts: US, −0.55 [95% CI, −0.77 to −0.34] vs −0.18 [95% CI, −0.25 to −0.10]; Europe, −1.49 [95% CI, −1.72 to −1.27] vs −0.24 [95% CI, −0.35 to −0.13]; and England, −0.48 [95% CI, −0.89 to −0.08] vs −0.23 [95% CI, −0.38 to −0.07]). This decreasing trend was more pronounced among women than men (point estimate in 1944-1948 for women vs men: US, −0.55 [95% CI, −0.86 to −0.30] vs −0.48 [95% CI, −0.84 to −0.13]; Europe, −1.50 [95% CI, −1.80 to −1.21] vs −1.34 [95% CI, −1.70 to −0.99]; and England, −0.76 [95% CI, −1.30 to −0.23] vs −0.07 [95% CI, -0.69 to 0.54]); the point estimates for men in England were not statistically significant.

**Conclusions and Relevance:**

In this cross-sectional study examining generational differences in age-specific dementia prevalence across the US, Europe, and England, we found that individuals from more recent birth cohorts had lower proportions of people with dementia. Work of this kind is relevant to plan for health and long-term care policy and workforce requirements across these regions now and into the future.

## Key Points

**Question:**

Does dementia prevalence differ across birth cohorts in the US, Europe, and England after accounting for age and period effects?

**Findings:**

In this cross-sectional study of 99 420 individuals in the US, 21 069 individuals in Europe, and 32 490 individuals in England, individuals from more recent-born birth cohorts had lower age-specific dementia prevalence rates in the US (21.2%), Europe (38.9%), and England (28.3%). This trend was more pronounced among women than men.

**Meaning:**

The generational decrease in dementia risk has important implications for health care planning, long-term care policies, and workforce requirements in aging populations.

## Teaser

This cross-sectional study estimates generational differences in age-specific dementia prevalence across the US, Europe, and England after accounting for age and period.

## Introduction

Dementia contributes significantly to disability and dependency among older people worldwide and was ranked as the seventh leading cause of death in 2023.^1^ It is estimated that there will be 152.8 million (95% CI, 130.8 million to 175.9 million) individuals living with dementia by 2050, with nearly 10 million incident cases annually.^2,3^ In 2019, the global economic burden of dementia was estimated to have reached 1.3 trillion US dollars (USD) and is predicted to reach 2.8 trillion USD by 2030.^1,4^ Dementia is already recognized as a key public health concern, but it is also a problem that is likely to increase in the future as average life expectancy continues to increase.

Understanding differences in age-specific dementia prevalence rates across regions, how rates have developed over time, and what this means for future prevalence estimates is an important consideration for accurately planning future health service needs. Different projections of current dementia prevalence rates and their trends exist in the literature. Some studies predict a decreasing trend in age-specific incidence rates,^5,6,7,8^ while others come to the opposite conclusion, predicting that age-specific incidence rates will increase.^9,10^ In a third group of studies, a stable trend in dementia incidence is predicted.^11,12^ Most of these studies, however, use data from 2010 onward, and most studies have focused on overall incidence rates, neglecting cohort-specific trends.^13,14^ In addition, cross-country comparisons have traditionally been very difficult due to the unavailability of suitable data and variations between countries regarding the types of data that have been collected.

Our study contributes to the extant literature by examining the association of cohort with age-specific dementia prevalence rates over time and across countries. We do so by using individual-level survey data with internationally coordinated questionnaires of older people living in the US, Europe, and England and applying an age-period-cohort methodology to disentangle generational differences in age-specific dementia prevalence across these regions.

There are several reasons why the consideration of possible cohort effects may be important when studying trends in dementia prevalence over time and for predicting future health needs.^15^ First, while age is a key factor associated with the onset of dementia, differences in the overall prevalence of dementia over time cannot be explained by age alone.^16^ Second, population health has been improving consistently over the last century, as indicated by sizable increases in life expectancy for recent cohorts compared with their forebears.^17^ Third, individuals in earlier birth cohorts arguably were born and raised in a very different context than the most recent birth cohorts, and in ways that may alter lifelong cognitive functioning, such as 2 world wars, economic recession, and improvements in educational systems, as well as health care delivery.^18^ Not considering cohort effects when studying prevalence estimates will therefore neglect any of these changes in modifiable risk factors across birth cohorts.

Studying cohort effects in dementia prevalence across regions is not, however, a straightforward undertaking. The Global Burden of Disease report^19^ points out the potential bias due to heterogeneity in cross-country comparisons, such as different data sources, the application of different diagnostic criteria or practices, and different dementia identification algorithms. The latter is crucial because identifying people with dementia in survey data is challenging because self-reports are usually underestimates of the true prevalence^20^ and diagnostic test results are generally not included in survey data.

Our study overcomes these challenges by studying the association of cohort with dementia using 3 comparable datasets for the US, Europe, and England and the same validated prediction algorithm to identify people with dementia. More specifically, we use data from the United States Health and Retirement Study (HRS), the Survey of Health, Ageing and Retirement in Europe (SHARE), and the English Longitudinal Study of Ageing (ELSA). These 3 longitudinal (panel) datasets are large and well-established population samples, which enable fairly precise estimates of the parameters of interest. All 3 databases contain similar data on demographic characteristics, educational attainment, and cognitive function, which are crucial variables for estimating dementia status.

## Methods

### Study Design and Data Sources

This study was designed and reported in accordance with the Strengthening the Reporting of Observational Studies in Epidemiology (STROBE) reporting guideline for cross-sectional studies. For our empirical analysis, we used individual, unit-record data from these 3 comparable nationally representative panel surveys, the HRS, the SHARE and the ELSA. The 3 surveys collect biennial data of individuals aged 51 years or older in the US (HRS) and individuals aged 50 years or older in Europe (SHARE) and England (ELSA).^21,22,23^ Our research was exempted from review and the requirement for informed consent was waived by The University of Queensland institutional review board because the data were deidentified.

From the HRS, we used waves 2 (1994) to 15 (2020-2021). From ELSA, we used waves 1 (2002-2003) to 9 (2018-2019). From SHARE, we used waves 1 to 2 (2004-2005 and 2006-2007) and waves 4 to 8 (2010-1011, 2013, 2015, 2017, and 2019-2020). For SHARE, wave 3 was not included in our study because it focuses on retrospective life histories of individuals^23^ and does not include important information collected in other waves. Only countries that were included in SHARE for all waves were chosen due to the data requirements of our model (this includes 10 European countries: Austria, Germany, Sweden, the Netherlands, Spain, Italy, France, Denmark, Switzerland, and Belgium). As was done by Hurd and colleagues,^24^ we focused on respondents older than 70 years of age. Summary statistics are presented in Table 1 and eTable 5 in Supplement 1.

**Table 1.** Summary Statistics

Variable^a^	Individuals, No. (%)		
	HRS (n = 99 420)	SHARE (n = 83 580)	ELSA (n = 31 384)
Gender			
Female	58 883 (59.2)	45 722 (54.7)	17 502 (55.8)
Male	40 537 (40.8)	37 858 (45.3)	13 882 (44.2)
Age group, y			
71-75	34 246 (34.4)	30 946 (37.0)	12 039 (38.4)
76-80	28 691 (28.9)	24 463 (29.3)	9068 (28.9)
81-85	20 179 (20.3)	16 363 (19.6)	6084 (19.4)
86-90	11 022 (11.1)	8449 (10.1)	3072 (9.8)
91-95	4184 (4.2)	2822 (3.4)	968 (3.1)
≥96	1098 (1.1)	537 (0.6)	153 (0.5)
Cohort years			
1890-1913	8858 (8.9)	NA	NA
1914-1918, 1901-1918, and 1908-1918	10 377 (10.4)	1497 (1.8)	1528 (4.9)
1919-1923	17 413 (17.5)	4470 (5.3)	3241 (10.3)
1924-1928	16 812 (16.9)	10 881 (13.0)	5116 (16.3)
1929-1933	16 434 (16.5)	17 751 (21.2)	7350 (23.4)
1934-1938	15 937 (16.0)	20 467 (24.5)	7564 (24.1)
1939-1943	10 298 (10.4)	19 963 (23.9)	4533 (14.4)
1944-1948	3291 (3.3)	8551 (10.2)	2052 (6.5)

Abbreviations: ELSA, English Longitudinal Study of Ageing; HRS, United States Health and Retirement Study; NA, not applicable; SHARE, Survey of Health, Ageing and Retirement in Europe.

^a^ Variable definitions are provided in eTable 5 in Supplement 1.

### Age Groups and Birth Cohorts

To study the association of age and cohort with dementia status, we divided the participants of the 3 surveys into 6 age groups, and 8 birth cohorts in the HRS as well as 7 birth cohorts in SHARE and ELSA. The first birth cohort for the HRS is from 1890 to 1913, the first cohort for SHARE is from 1901 to 1918, and the first cohort for ELSA is from 1908 to 1918, which we used as reference categories in our cohort analyses. eTable 1, eTable 2, and eTable 3 in Supplement 1 present sample sizes for all birth-age-cohort combinations for each survey. Table 2 provides an overview of age group, cohort, and year combinations.

**Table 2.** An Overview of Age Group, Cohort, and Year Combinations^a^

Age, y	1994	1999	2004	2009	2014	2019
71-75	1919-1923	1924-1928	1929-1933	1934-1938	1939-1943	1944-1948
76-80	1914-1918	1919-1923	1924-1928	1929-1933	1934-1938	1939-1943
81-85	1909-1913	1914-1918	1919-1923	1924-1928	1929-1933	1934-1938
86-90	1904-1908	1909-1913	1914-1918	1919-1923	1924-1928	1929-1933
91-95	1899-1905	1904-1908	1909-1913	1914-1918	1919-1923	1924-1928
≥96	<1899	<1904	<1909	<1914	<1919	<1924

^a^ The United States Health and Retirement Study survey began in 1992 and is thus much larger than the Survey of Health, Ageing and Retirement in Europe and English Longitudinal Study of Ageing, which started in 2002 and 2004, respectively, resulting in more birth cohorts participating in the United States Health and Retirement Study survey.

### Statistical Analysis

#### Identifying Respondents With Dementia

Statistical analysis was performed from May 2023 to February 2025. To estimate each respondent’s dementia status, we followed a validated algorithm^24^ and used findings from the Aging, Demographics, and Memory Study (ADAMS) to estimate probable dementia among respondents in the HRS, SHARE, and ELSA. The ADAMS is a substudy of the HRS, which included a subset of 856 participants older than 70 years who provided data for the 2000 or 2002 waves of the HRS. The respondents underwent an extensive in-home cognitive assessment lasting 3 to 4 hours. A panel of experts reached a consensus regarding the final diagnosis of dementia based on the *Diagnostic and Statistical Manual of Mental Disorders* (Third Edition) criteria and the *Diagnostic and Statistical Manual of Mental Disorders* (Fourth Edition) criteria.^25,26,27^ As other studies have done,^28,29^ we slightly modified and then applied the algorithm to the SHARE and ELSA to identify respondents with dementia in these datasets. The algorithm that we applied has been validated and used in previous studies, demonstrating maximized accuracy compared with other commonly used algorithms.^25^

The algorithm used ordered probit models to estimate the likelihood of dementia based on respondent’s demographic characteristics, number of limitations with (instrumental) activities of daily living, and cognitive functioning scores, as well as the changes in how individuals scored on these tests between survey waves. If a respondent’s limitations prevented them from answering the questions directly, proxy replies were used. Proxy respondents were primarily relatives or in some cases paid helpers, professionals, or other caregivers. The HRS, SHARE, and ELSA assess cognitive function via the Telephone Interview for Cognitive Status for self-respondents and the Informant Questionnaire on Cognitive Decline in the Elderly for participants represented by proxy. We adjusted the algorithm slightly for SHARE and ELSA respondents because some of the questions used in the algorithm from the HRS were not asked in these surveys. We did not include variables that were not included in SHARE or ELSA in some or all waves (see equations 1, 2, and 3 in the eMethods in Supplement 1). We tested the validity of our slightly adapted algorithms for SHARE and ELSA by comparing people’s estimated dementia status using the respective algorithms with their clinically confirmed dementia status in the ADAMS. The validity of the algorithms is 85.9% for the HRS and 85.7% for SHARE and ELSA. Detailed information about all variables used in the algorithm by survey is presented in eTable 4 in Supplement 1, with summary statistics in eTable 5 and eTable 6 in Supplement 1 providing an overview of the coefficients used in the algorithms.

As a robustness check, we used a machine learning algorithm to estimate dementia status among survey respondents. The multilayer perceptron model^30^ is one of the more traditional machine learning methods. For our models, we used 1 hidden layer and a rectified linear unit^31^ as the activation function (see equation 4 in the Methods in Supplement 1). We also invoked an early stopping mechanism to avoid overfitting. The variables that we used to estimate dementia status are the same as those in the main analysis. The model performances are evaluated by comparing the dementia status predicted by the models with the status in the dataset (confirmed by clinical professionals). The resultant models all achieved estimation accuracy over 85% for both types of participants (ie, proxy and nonproxy). The validation accuracies for each category are as follows: (1) HRS self-respondents, 86.4%; (2) HRS proxy, 87.5%; (3) SHARE self-respondents, 87.1%; (4) SHARE proxy respondents, 92.5%; (5) ELSA self-respondents, 87.1%; and (6) ELSA proxy respondents, 92.5%. The definition of validity was the same as the algorithm in the main analysis (ie, the percentage of respondents for whom predicted status is equal to the “true” status as determined in the HRS substudy).

#### Estimating Association of Cohort With Dementia Status

A large, methodological literature exists that explains the problem of studying cohort effects given the coexistence of possible age and period effects. Although it is possible to control for age and cohort effects in the same equations, it becomes problematic if one also wants to control for period effects. Not including period effects may, however, bias estimates because specific interventions or events that happen in a given year may affect the health of all ages and cohorts. The problem in controlling for age, period, and cohort lies in the linear association between the 3 variables, where age = year − birth year.

One way to overcome the linearity problem is to use a proxy for 1 of the 3 variables.^32^ Because we are interested in estimating cohort effects and consider age an important determinant of the onset of dementia, we decided to use year effects as a proxy. There is a definite and strong positive correlation between the gross domestic product (GDP) and health. Several articles have used GDP growth rates as proxy variables for period effects.^33,34,35,36^ The Preston curve describes the association between income and health status, whereby nations with higher incomes tend to be healthier than nations with lower incomes.^37^ We followed the literature and used the GDP growth rate of the US, our selected European countries, and England^38,39^ as a proxy for period effects and incorporated random effects in our statistical analysis. This allowed us to control for periods of economic downturns, which have been closely associated with health.^37^

The generalized linear mixed models that we used to estimate the association of cohort with dementia status are as follows: (1) dementia*_i,t_* = δ_0 _+ λ_1_age group*_a,i,t_* + λ_2_cohort*_c,i_* + λ_3_year*_i,t_* + *ϵ_i,t_* and (2) dementia*_i,t_* = δ_1_ + μ_1_age group*_a,i,t_* + μ_2_cohort*_c,i_* + μ_3_GDP growth rate*_i,t_* + *ϵ_i,t_*, where *a* represent the age groups (1 indicates age of 71-75 years; 2, age of 76-80 years; 3, age of 81-85 years; …6, age of ≥95 years); *i* denotes individuals; *t* denotes the current survey year; and *c* indicates the birth cohorts from the HRS, SHARE, and ELSA. GDP growth rate*_i_*_,_*_t_* is the GDP growth rate in the current survey year for each respective country.

We used Stata, version 17.0 (StataCorp LLC) and Python, version 3.11.7 (Python Software Foundation) to estimate dementia status for individuals; Stata, version 17.0 to estimate the association of cohort with dementia status; and Stata, version 17.0 and R, version 4.4.0 (R Project for Statistical Computing) for data visualization. All *P* values were from 2-sided tests and results were deemed statistically significant at *P* < .05. We reported 95% CIs for all estimates.

## Results

**Figure 1.**
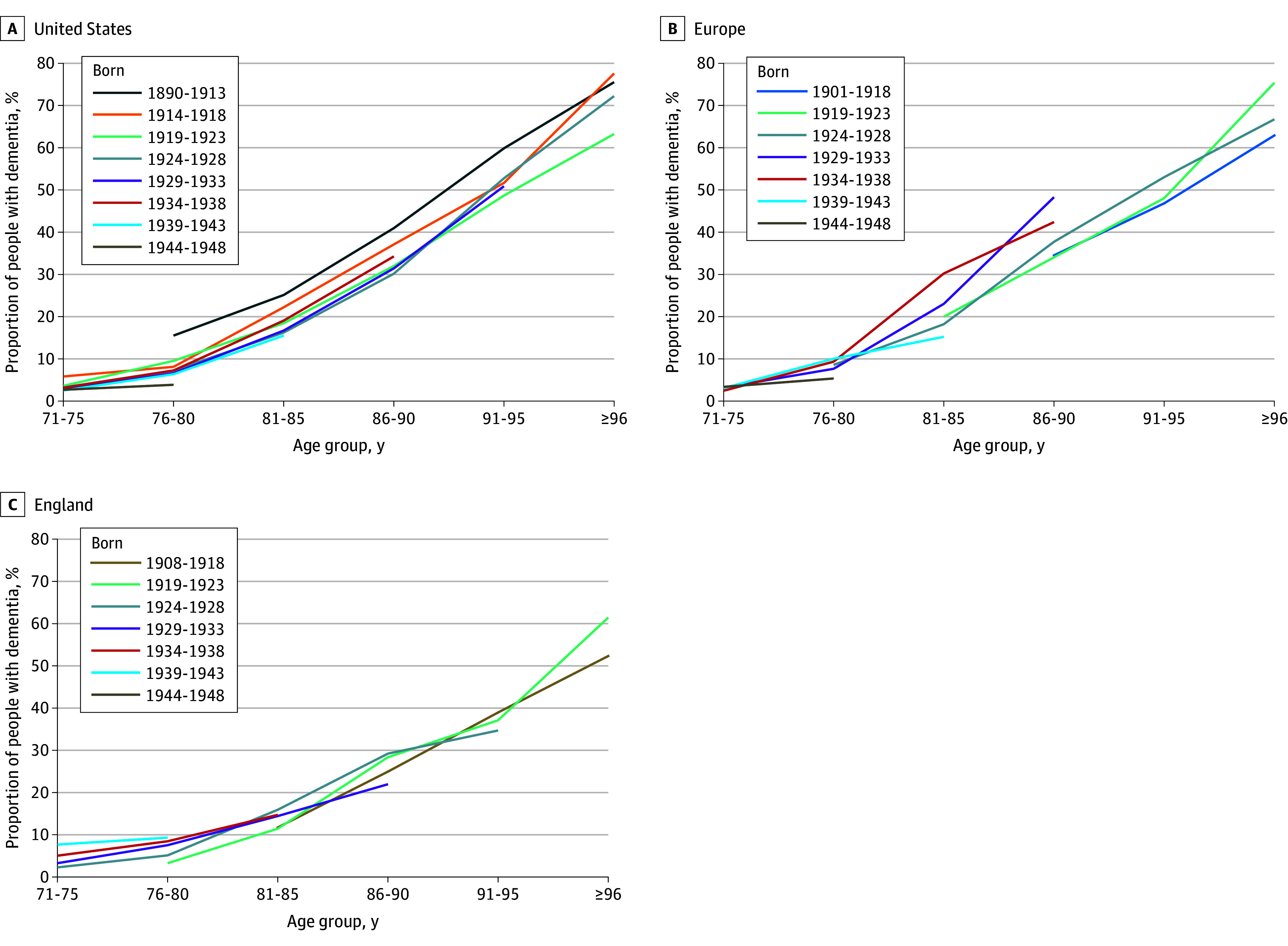
Mean Prevalence of Dementia by Age Groups and Birth Cohorts in the US, Europe, and England Source: Based on authors’ calculation from the United States Health and Retirement Study; Survey of Health, Ageing and Retirement in Europe; and English Longitudinal Study of Ageing datasets.

Of the 99 420 individuals (mean [SD] age, 79.1 [6.2] years; 59.2% women) in the HRS dataset, 21 069 (21.2%) were included in this study; of the 83 580 individuals (mean [SD] age, 78.6 [5.9] years; 54.7% women) in the SHARE dataset, 32 490 (38.9%) were included in this study; and of the 31 384 individuals (mean [SD] age, 77.9 [5.6] years; 55.8% women) in the ELSA dataset, 8878 (28.3%) were included in this study. Figure 1 shows the trends in the mean prevalence of dementia by age groups and birth cohorts in the US, Europe, and England, respectively. As expected, the prevalence of dementia increased by age among all birth cohorts in all 3 regions, suggesting that, at a given age, respondents born more recently seemed to include a lower proportion of people with dementia compared with those born in earlier birth cohorts. For respondents aged 81 to 85 years, the proportion of people who developed dementia varied across birth cohorts and regions. In the US, 25.1% of individuals born between 1890 and 1913 developed dementia compared with 15.5% of those born between 1939 and 1943. In Europe, 30.2% of individuals born between 1934 and 1938 developed dementia compared with 15.2% of those born between 1939 and 1943. In England, 15.9% of individuals born between 1924 and 1928 developed dementia compared with 14.9% of those born between 1934 and 1938 did.

Table 3 shows the estimated association of cohort with dementia status among respondents after controlling for age and period (GDP growth rates). eTable 7 and eTable 8 in Supplement 1 show the results for the estimated prevalence of dementia in the US, Europe, England overall and for women and men. eTable 9 and eTable 10 in Supplement 1 show the association of cohort with dementia status across the regions for women and men, respectively. eTable 11, eTable 12, and eTable 13 in Supplement 1 show the association of cohort with dementia status in all 3 regions controlling for age and period effects (without GDP growth rates). The results from models with and without GDP growth rates were consistent. For the combined population, the results were statistically significant, indicating that generational differences play an important role in determining dementia prevalence rates. We found that generations born more recently were less likely to have dementia at a given age compared with their respective reference cohort, which is consistent with some results produced previously.^11,40^ In the US, Europe, and England, the 1919-1923 cohort was negatively associated with dementia at the 1% level, indicating a consistent and strong decreasing trend in the proportion of people with dementia for these cohorts at a given age, with the effect becoming more pronounced in later cohorts (cohort effect for 1944-1948 vs 1919-1923 cohorts: US, −0.55 [95% CI, −0.77 to −0.34] vs −0.18 [95% CI, −0.25 to −0.10]; Europe, −1.49 [95% CI, −1.72 to −1.27] vs −0.24 [95% CI, −0.35 to −0.13]; and England, −0.48 [95% CI, −0.89 to −0.08] vs −0.23 [95% CI, −0.38 to −0.07]) (Table 3).

**Table 3.** Estimated Association of Cohort With Dementia Status in the US, Europe, and England

Cohort	Estimated coefficient (95% CI)^a^		
	US	Europe	England
1919-1923	−0.18 (−0.25 to −0.10)^b^	−0.24 (−0.35 to −0.13)^b^	−0.23 (−0.38 to −0.07)^b^
1924-1928	−0.27 (−0.37 to −0.18)^b^	−0.45 (−0.57 to −0.33)^b^	−0.33 (−0.53 to −0.14)^b^
1929-1933	−0.32 (−0.44 to −0.21)^b^	−0.61 (−0.76 to −0.47)^b^	−0.49 (−0.72 to −0.25)^b^
1934-1938	−0.31 (−0.45 to −0.17)^b^	−0.84 (−1.08 to −0.67)^b^	−0.52 (−0.82 to −0.22)^b^
1939-1943	−0.46 (−0.63 to −0.29)^b^	−1.19 (−1.38 to −0.99)^b^	−0.45 (−0.80 to −0.10)^c^
1944-1948	−0.55 (−0.77 to −0.34)^b^	−1.49 (−1.72 to −1.27)^b^	−0.48 (−0.89 to −0.08)^c^
No. of observations	99 420	83 580	31 384

^a^ In all regressions we controlled for age groups and gross domestic product growth rate for each region. The 95% CIs in parentheses are clustered at the individual level.

^b^ *P* < .01.

^c^ *P* < .05.

For our female sample, we found that the results were statistically significant in all 3 regions. Similar to the results that we obtained for the whole sample, women born more recently in the US, Europe, and England were less likely to have dementia (point estimate for 1944-1948 vs 1919-1923 cohorts: US, −0.58 [95% CI, −0.86 to −0.30] vs −0.21 [95% CI, −0.30 to −0.11]; Europe, −1.50 [95% CI, −1.80 to −1.21] vs −0.23 [95% CI, −0.37 to −0.09]; and England, −0.76 [95% CI, −1.30 to −0.23] vs −0.31 [95% CI, −0.50 to −0.11]). In England, the results for men were not statistically significant (point estimate for 1919-1923, –0.07 [95% CI, –0.33 to 0.20]; for 1924-1928, –0.15 [95% CI, –0.46 to 0.16]; for 1929-1933, –0.32 [95% CI, –0.69 to 0.06]; for 1934-1938, –0.17 [95% CI, –0.65 to 0.31]; for 1939-1943, –0.06 [95% CI, –0.61 to 0.48]; and for 1944-1948, –0.07 [95% CI, –0.69 to 0.54]). The younger male generations in the US and Europe were less likely to have dementia compared with the previous generations (point estimate for 1944-1948 vs 1919-1923 cohorts: US, −0.48 [95% CI, −0.84 to −0.13] vs −0.10 [95% CI, −0.23 to 0.20]; Europe, −1.34 [95% CI, −1.70 to −0.99] vs −0.19 [95% CI, −0.38 to −0.01]; and England, −0.07 [95% CI, −0.69 to 0.54] vs −0.07 [95% CI, −0.33 to 0.20]). The decreasing trends among women in all 3 regions have been significantly higher than that of men (point estimate in 1944-1948 for women vs men: US, −0.55 [95% CI, −0.86 to −0.30] vs −0.48 [95% CI, −0.84 to −0.13]; Europe, −1.50 [95% CI, −1.80 to −1.21] vs −1.34 [95% CI, −1.70 to −0.99]; and England, −0.76 [95% CI, −1.30 to −0.23] vs −0.07 [95% CI, –0.69 to 0.54]). These results suggest that the decreasing trend in prevalence among women plays an important role in explaining the decrease of age-specific dementia incidence rates.

**Figure 2.**
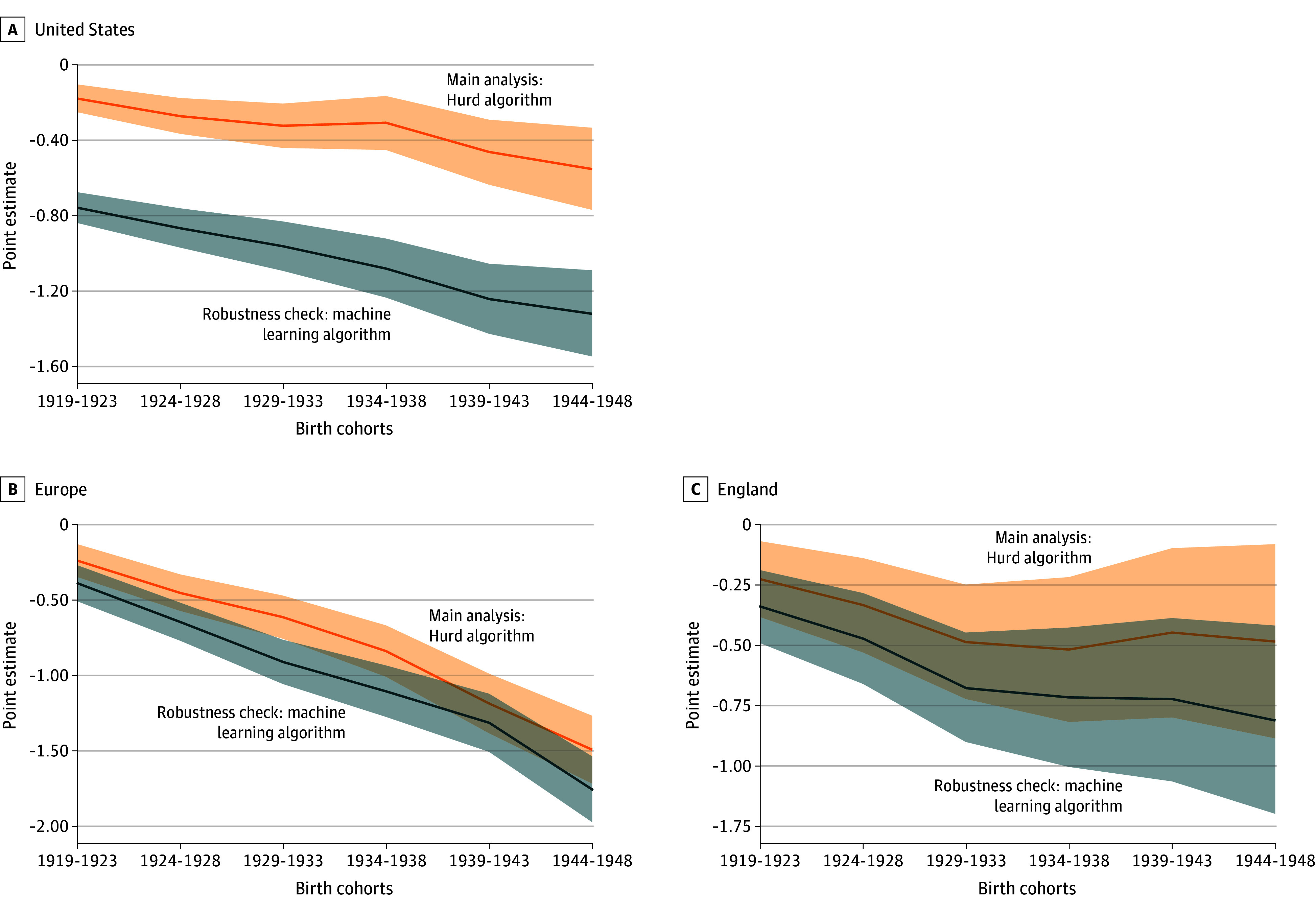
Association of Cohort With Dementia When Controlling for Age Groups and Period in the US, Europe, and England A comparison between the main analyses and robustness checks is shown. The association of cohort with dementia for the US, Europe, and England are estimated using data from the United States Health and Retirement Study; the Survey of Health, Ageing and Retirement in Europe; and the English Longitudinal Study of Ageing. The reference group is the birth cohort (1890-1918, 1901-1923, and 1908-1918, respectively). Shaded areas represent 95% CIs.

The results of our robustness analysis using GDP growth rate as proxy for year effects are displayed in eTable 14 in Supplement 1. The trends are consistent with the results of the main analysis, showing statistically significant effects. Direct comparisons between the 2 methods are displayed in Figure 2. The robustness check suggests that our results provide robust evidence of negative associations, where people born more recently were less likely to have dementia in the US, Europe, and England (eTable 15 and eTable 16 in Supplement 1). eTable 17 in Supplement 1 shows the details of missing data for key variables.

## Discussion

In this cross-sectional analysis, we used data from 3 comparable surveys from the US, Europe, and England to estimate how the prevalence of dementia has changed across birth cohorts after controlling for age and period. Our research is crucial for understanding generational trends in dementia prevalence, providing guidance for health policymakers to address shifting risks and health care needs over time. We found that, compared with people born earlier, people born more recently, in all 3 regions, have lower proportions of dementia. Our study thus shows decreasing age-specific prevalence trends in the US, Europe, and England.

These findings contribute to the literature in several important ways. To our knowledge, we are the first to consider 3 highly comparable datasets that allow cross-country comparison of dementia prevalence rates, using a validated algorithm that can be applied to identify respondents with dementia. To ensure the robustness of our estimate, we also used modern machine learning techniques, confirming the validity of the estimated dementia status among respondents. Our results provide confidence that potential measurement error due to heterogeneity in different databases and algorithms is overcome. Our method addresses the underestimation of prevalence rates when using solely medical records or self-reports to identify people with dementia. Especially when studying cohort effects, it is of utmost importance that prevalence estimates are not biased due to changes in clinical practice over time.

Second, we improve on previous applications by overcoming potential dementia identification issues that have been raised recently for a study in England.^41^ More specifically, we only used variables that are available across all waves in the respective surveys to estimate participants’ dementia status. This will mitigate any problems that may arise due to missing observations.

Third, we provide additional evidence on estimating cohort effects in dementia prevalence. Similar to earlier studies estimating generational differences when estimating trends in dementia incidence and prevalence rates, our analysis captures aspects that age and period effects may not fully account for. For example, people born at the beginning of the 20th century witnessed 2 world wars, with far-reaching social and economic consequences. In the 1950s, industrialization and urbanization resulted in increased exposure to pollutants and toxins that are all found to play a significant role in predicting dementia risk and that may affect people in different phases of their life differently, captured in the cohort effects.

Fourth, we provide new evidence on gender disparities in the prevalence of dementia. Our findings suggest that the gender disparities in age-specific dementia prevalence narrowed in these 3 regions over the past decades, but the prevalence difference between genders remains notable. One explanation for this trend could be the increased educational attainment among women and girls compared with previous decades, one of the key modifiable risk factors of dementia.^42^ However, substantial gender disparities persist, potentially due to historical and societal factors that have disproportionately affected womens’ overall well-being and access to resources over a long period.

### Limitations

Our study has some limitations. First, our research is limited by data availability. For example, SHARE lacks wave 3 (2008), and we adjusted the algorithm when applying to the SHARE and ELSA data because of the lack of variables across some waves. Second, we may underestimate the prevalence of dementia as some respondents may have had the disease and died between survey waves. Third, there may be biases in database sampling, such as lower-than-proportional representation of individuals from racial and ethnic minority groups in ELSA. Fourth, the potential effect of data collection methods is crucial in estimating dementia prevalence trends, which we are unable to validate in this retrospective analysis. Fifth, as previous literature has suggested, the current mainstream 12 risk factors for dementia can explain only 40% of dementia prevalence.^16^ Although our study estimates the generational differences in dementia prevalence, it does not investigate the underlying reasons for the decrease, which will be an important consideration for future studies.

## Conclusions

In this cross-sectional study of age-specific dementia prevalence rates across cohorts, we provided evidence of decreasing trends in the US, Europe, and England. Our findings were consistent with most recent studies in high-income regions that had indicated a decrease in the prevalence of dementia^9,10^ and studies that had claimed reduced incidence or prevalence with later birth cohorts.^8,43,44,45,46^ If such trends continue, the health and socioeconomic burden of dementia is not likely to increase in the future beyond the burden associated with population aging.^47^ Our research is crucial for informing public health policies on expenditures and interventions aimed at preventing or managing dementia effectively across different generations, genders, and regions, not just age groups. There is a strong need to continue to observe trends in dementia to prepare for future health planning.

## Notes

Supplement 1. **eTable 1.** Sample Sizes for HRS Survey Periods 1992-2022 Across Age Groups and Birth Cohort **eTable 2.** Sample Sizes for SHARE Survey Periods 2004-2020 Across Age Groups and Birth Cohorts **eTable 3.** Sample Sizes for the ELSA Survey Periods 2002-2019 Across Age Groups and Birth Cohorts **eMethods.** Identifying Respondents With Dementia **eTable 4.** Variable Deﬁnitions and Availability **eTable 5.** Summary Statistics for Variables Used in the Algorithm **eTable 6.** Predictive Algorithms **eTable 7.** Estimated Prevalence of Dementia in the United States, Europe, England **eTable 8.** Estimated Prevalence of Dementia for Females and Males in the US, Europe and England **eTable 9.** Cohort Effects Estimated for Females in the US, Europe and England **eTable 10.** Cohort Effects Estimated for Males in the US, Europe and England **eTable 11.** Cohort Effects Estimated in the US, Europe and England **eTable 12.** Cohort Effects Estimated for Females in the US, Europe and England **eTable 13.** Cohort Effects Estimated for Males in the US, Europe and England **eTable 14.** Robustness Check Results of Age, Cohort, Period for Individuals in the US, Europe and England **eTable 15.** Robustness Check Results of Age, Cohort, Period for Females in the US, Europe and England **eTable 16.** Robustness Check Results of Age, Cohort, Period for Males in the US, Europe and England **eTable 17.** Summary of Missing Data for Key Variables
eReference.

Supplement 2.
Data Sharing Statement
