# Supplementary material for: Generational Differences in Age-Specific Dementia Prevalence Rates
Source: JAMA Netw Open. 2025 Jun 2;8(6):e2513384. doi: 10.1001/jamanetworkopen.2025.13384 (PMC12551361; doi:10.1001/jamanetworkopen.2025.13384)
Supplement: Supplement 1. — eTable 1. Sample Sizes for HRS Survey Periods 1992-2022 Across Age Groups and Birth Cohort eTable 2. Sample Sizes for SHARE Survey Periods 2004-2020 Across Age Groups and Birth Cohorts eTable 3. Sample Sizes for the ELSA Survey Periods 2002-2019 Across Age Groups and Birth Cohorts eMethods. Identifying Respondents With Dementia eTable 4. Variable Deﬁnitions and Availability eTable 5. Summary Statistics for Variables Used in the Algorithm eTable 6. Predictive Algorithms eTable 7. Estimated Prevalence of Dementia in the United States, Europe, England eTable 8. Estimated Prevalence of Dementia for Females and Males in the US, Europe and England eTable 9. Cohort Effects Estimated for Females in the US, Europe and England eTable 10. Cohort Effects Estimated for Males in the US, Europe and England eTable 11. Cohort Effects Estimated in the US, Europe and England eTable 12. Cohort Effects Estimated for Females in the US, Europe and England eTable 13. Cohort Effects Estimated for Males in the US, Europe and England eTable 14. Robustness Check Results of Age, Cohort, Period for Individuals in the US, Europe and England eTable 15. Robustness Check Results of Age, Cohort, Period for Females in the US, Europe and England eTable 16. Robustness Check Results of Age, Cohort, Period for Males in the US, Europe and England eTable 17. Summary of Missing Data for Key Variables eReference. [file jamanetwopen-e2513384-s001.pdf]

Supplementary Online Content

Dou X, Lenzen S, Connelly LB, Lin R. Generational differences in age-specific dementia prevalence rates. *JAMA Netw Open.* 2025;8(6):e2513384.
doi:10.1001/jamanetworkopen.2025.13384

eTable 1. Sample Sizes for HRS Survey Periods 1992-2022 Across Age Groups and Birth Cohort
eTable 2. Sample Sizes for SHARE Survey Periods 2004-2020 Across Age Groups and Birth Cohorts
eTable 3. Sample Sizes for the ELSA Survey Periods 2002-2019 Across Age Groups and Birth Cohorts
eMethods. Identifying Respondents With Dementia
eTable 4. Variable Definitions and Availability
eTable 5. Summary Statistics for Variables Used in the Algorithm
eTable 6. Predictive Algorithms
eTable 7. Estimated Prevalence of Dementia in the United States, Europe, England
eTable 8. Estimated Prevalence of Dementia for Females and Males in the US, Europe and England
eTable 9. Cohort Effects Estimated for Females in the US, Europe and England
eTable 10. Cohort Effects Estimated for Males in the US, Europe and England
eTable 11. Cohort Effects Estimated in the US, Europe and England
eTable 12. Cohort Effects Estimated for Females in the US, Europe and England
eTable 13. Cohort Effects Estimated for Males in the US, Europe and England
eTable 14. Robustness Check Results of Age, Cohort, Period for Individuals in the US, Europe and England
eTable 15. Robustness Check Results of Age, Cohort, Period for Females in the US, Europe and England
eTable 16. Robustness Check Results of Age, Cohort, Period for Males in the US, Europe and England
eTable 17. Summary of Missing Data for Key Variables
eReference.

This supplementary material has been provided by the authors to give readers additional information about their work.

© 2025 Dou X et al. *JAMA Network Open.*

Sample size

eTable 1. Sample Sizes for HRS Survey Periods 1992-2022 Across Age Groups and Birth Cohort

	Age groups						
	71-75	76-80	81-85	86-90	91-95	96 +	Total
1890-1913	NA	355	2 876	3 163	1 790	674	8 858
1914-1918	482	3 746	3 206	1 964	747	232	10 377
1919-1923	5 304	5 018	3 770	2 163	984	174	17 413
1924-1928	5 705	4 910	3 440	2 131	608	18	16 812
1929-1933	6 252	4 958	3 682	1 487	55	NA	16 434
1934-1938	7 006	5 812	3 005	114	NA	NA	15 937
1939-1943	6 363	3 735	200	NA	NA	NA	10 298
1944-1948	3 134	157	NA	NA	NA	NA	3 291
Total	34 246	28 691	20 179	11 022	4 184	1 098	99 420

© 2025 Dou X et al. *JAMA Network Open.*

eTable 2. Sample Sizes for SHARE Survey Periods 2004-2020 Across Age Groups and Birth Cohorts

	Age groups						
	71-75	76-80	81-85	86-90	91-95	96 +	Total
1901-1918	NA	NA	NA	676	511	310	1 497
1919-1923	NA	NA	1 798	1 269	1 188	215	4 470
1924-1928	NA	3 124	2 861	3 822	1 062	12	10 881
1929-1933	3 942	4 274	6 929	2 545	61	NA	17 751
1934-1938	5 522	10 263	4 545	137	NA	NA	20 467
1939-1943	13 287	6 446	230	NA	NA	NA	19 963
1944-1948	8 195	356	NA	NA	NA	NA	8 551
Total	30 946	24 463	16 363	8 449	2 822	537	83 580

© 2025 Dou X et al. *JAMA Network Open.*

eTable 3. Sample Sizes for the ELSA Survey Periods 2002-2019 Across Age Groups and Birth Cohorts

	Age groups						
	71-75	76-80	81-85	86-90	91-95	96 +	Total
1908-1918	NA	NA	197	806	416	109	1 528
1919-1923	NA	372	1 552	917	356	44	3 241
1924-1928	497	2 094	1 504	825	196	NA	5 116
1929-1933	2 776	2 344	1 706	524	NA	NA	7 350
1934-1938	3 513	2 926	1 125	NA	NA	NA	7 564
1939-1943	3 201	1 332	NA	NA	NA	NA	4 533
1944-1948	2 052	NA	NA	NA	NA	NA	2 052
Total	12 039	9 068	6 084	3 072	968	153	31 384

© 2025 Dou X et al. *JAMA Network Open.*

eMethods. Identifying Respondents With Dementia

Main analysis for identifying respondents with dementia

For all non-proxy respondents, the model we use to predict dementia is as follows:

$$Dementia_{i,t} = \beta_1 TICS_{i,t} + \beta_2 \Delta TICS_{i,t} + \beta_3 X_{i,t} + \epsilon_{i,t} \quad (1)$$

where $Dementia_{i,t}$ is a binary variable taking on two values (= 1 if living with dementia, = 0 otherwise). *TICS* represents cognitive function scores from the TICS assessment in the current wave. $\Delta TICS_{i,t}$ denotes the change in the scores of cognitive function from the TICS assessment between the prior wave and current wave and $X_{i,t}$ are demographic characteristics including five-year age bands, education, gender, scores of ADL and IADL and their changes between the prior wave and the current wave. See variable definitions in eTable A4 and summary statistics in eTable A5.

To predict dementia for proxy respondents in HRS, we use the following model:

$$Dementia_{i,t} = \gamma_1 IQCODE_{i,t} + \gamma_2 \Delta IQCODE_{i,t} + \gamma_3 PRIORPROXY_{i,t} + \gamma_4 PRIORTICS_{i,t} + \gamma_5 X_{i,t} + \epsilon_{i,t} \quad (2)$$

where *IQCODE* indicates the score of the Jorm IQCODE in the current wave. $\Delta IQCODE_{i,t}$ represents the change between the prior wave and the current one. *PRIORPROXY* indicates whether the participant was a proxy respondent in the prior wave. *PRIORTICS* denotes the cognitive function from the TICS assessment in the prior wave. $X_{i,t}$ is defined as above.

To predict dementia among proxy respondents in SHARE and ELSA, the model is as follows:

$$Dementia_{i,t} = \theta_1 PRIORPROXY_{i,t} + \theta_2 PRIORTICS_{i,t} + \theta_3 X_{i,t} + \epsilon_{i,t} \quad (3)$$

Model (1) was estimated using an ordered probit model. There are two-stage prediction process. In the first stage, we estimate coefficients for each predictor variable using ADAMS data, where individual dementia status is known. This stage generates coefficients for demographics (age, gender, education, (I)ADL) and cognitive functioning measures (TICS, ΔTICS), separately for HRS, SHARE, and ELSA, based on the model applied to each dataset. Please see details in eTable 4-6 for the variable definitions, summary statistics and corresponding coefficients.

© 2025 Dou X et al. *JAMA Network Open.*

In the second stage, we apply these estimated coefficients to predict dementia status for all individuals in HRS, SHARE, and ELSA. The outcome variable is a binary measure of dementia status, determined by an underlying unobserved index variable, y* (cognitive functioning). Individuals with y* below cutoff c2 are classified as having dementia, while those with y* above cut-off c2 are classified as having normal cognitive functioning.

Robustness check for identifying respondents with dementia

ReLU is a popular activation function used in Multi-Layer Perceptrons (MLPs) and other neural networks. The values from the hidden layer are then forwarded to the output layer as the classification results. As this is a binary classification problem, we let the output layer have two neurons to represent two possibilities and use the cross-entropy loss as the loss function to evaluate model performance. To enable training process, we use Adam (1) as the optimization method with a learning rate of 0·001 and a batch size of 1024. We also invoke an early stopping mechanism to avoid overfitting.

We develop our models based on the ADAMS dataset, divided into a training set (70%) and a testing set (30%). The variables we used to predict dementia status are the same as those in the main analysis. The model performances are evaluated by comparing the dementia status predicted by the models with the status in the dataset (confirmed by clinical professionals). The resultant models all achieve estimation accuracy over 85% for both types of participants (i.e. proxy and non-proxy).

The model we use to predict dementia is as follows:

$$\boldsymbol{z}_i = \sigma\left(\boldsymbol{W}_{i-1,i}\boldsymbol{z}_{i-1} + \boldsymbol{b}_{i-1,i}\right) \tag{4}$$

where the output of the *i*th layer ($\mathbf{z}_i$) is obtained by passing the output of the previous layer ($\mathbf{z}_{i-1}$) through a predefined activation function (σ), along with a specific weight ($\mathbf{W}_{i-1,i}$) and bias ($b_{i-1,i}$). $\mathbf{W}_{i-1,i}$ and $b_{i-1,i}$ are adjustable parameters that are optimized during the training phase.

© 2025 Dou X et al. *JAMA Network Open.*

eTable 4. Variable Definitions and Availability

	Abbreviations	Definitions	HRS	SHARE	ELSA
Activity of Daily Living	ADL	A series questions to assess self- care tasks such as walking, eating, dressing and grooming, bathing, toileting, and transferring.	√	√	√
Instrumental Activity of Daily Living	IADL	A series questions to assess more complex activities, such as managing finances, navigating transportation, shopping and meal preparation, house cleaning and maintenance, medication management, and communication.	√	√	√
Date naming/ Orientation	dates	Reporting "today's date," which included the month, day, year, and day of the week, was requested of the respondents.	√	√	√
Backward count starting from 20	bwc20	The task required of the respondents was to count backward for ten consecutive numbers, starting with 20.	√	NA	NA
Serial 7's test	serial7	For a total of five trials, the interviewer asked the respondent to deduct 7 from 100 and then subtract 7 from each succeeding number.	√	NA	NA
Object naming	scissor / cactus	"What do you usually use to cut paper?" "What do you call the kind of prickly plant that grows in the desert?"	√	NA	NA
President naming	president	Respondents were asked to name the current President of the United States or Prime minister of the United Kingdom.	√	NA	NA
Immediate word recall	immediate recall	After reading the respondent a list of twenty nouns, the interviewer asked the respondent to recollect as many terms as they could, in any sequence.	√	√	√
Delayed word recall	delayed recall	The respondents were asked to recollect the nouns that had been previously provided as part of an instant recall test after another five minutes or so of asking survey questions.	√	√	√
Informant Questionnaire on Cognitive Decline in the Elderly	IQCODE	A validated tool with 16 questions to evaluate cognitive function for respondents who were represented by a proxy in the surveys.	√	NA	NA

© 2025 Dou X et al. *JAMA Network Open.*

eTable 5. Summary Statistics for Variables Used in the Algorithm

	HRS (N = 99 420)	SHARE (N=83 580)	ELSA (N=31 384)
**Educational attainment**			
High school degree	0·31 (0·46)	0·24 (0·43)	0·15 (0·35)
More than high school degree	0·36 (0·48)	0·20 (0·40)	0·27 (0·44)
**Physical functioning**			
Number of limitations with Activity of Daily Living	0·59 (1·23)	0·45 (1·14)	0·54 (1·08)
Number of limitations with Instrumental Activity of Daily Living	0·61 (1·30)	0·79 (1·50)	0·41 (1·03)
**Cognitive functioning tests**			
Date naming/orientation test	3·16 (1·40)	3·27 (1·35)	3·41 (1·14)
Backward count starting from 20	0·82 (0·38)	NA	NA
Serial 7's test	2·89 (1·95)	NA	NA
Object naming - scissor	0·86 (0·34)	NA	NA
Object naming - cactus	0·78 (0·42)	NA	NA
President naming	0·82 (0·38)	NA	NA
Immediate wordrecall	3·62 (2·51)	4·28 (1·93)	4·70 (2·15)
Delayed wordrecall	2·96 (2·32)	2·86 (2·11)	3·30 (2·25)
Informant Questionnaire on Cognitive Decline in the Elderly	0·38 (1·16)	NA	NA

Note: N = Number of observations. Data are presented in the form: mean (SD). Summary statistics are for HRS, SHARE and ELSA participants aged more than 70 years of age.

© 2025 Dou X et al. *JAMA Network Open.*

**eTable 6.** Predictive Algorithms				
	HRS	HRS proxy	ELSA	SHARE
age75-79	-0·20	-0·11	-0·21	-0·43
age80-84	-0·55	-1·10	-0·50	-0·96
age85-89	-0·65	-1·15	-0·64	-0·95
age90plus	-0·87	-0·78	-0·82	-0·68
High schoolgraduation	-0·39	0·18	-0·23	-0·02
More than high school	-0·29	0·21	-0·08	-0·13
female	-0·13	-0·34	-0·24	-0·37
ADL	0·08	-0·17	0·07	-0·02
IADL	-0·26	-0·31	-0·26	-0·53
ADL change	-0·11	0·21	-0·09	0·12
IADL change	0·16	0·15	0·10	0·14
dates	0·34	0·00	0·38	0·00
bwc20	-0·44	NA	NA	NA
serial7	0·15	0·00	NA	NA
scissor	-0·72	NA	NA	NA
cactus	0·00	NA	NA	NA
president	0·33	0·00	NA	NA
immediate recall	0·20	0·00	0·21	0·00
delayed recall	0·24	0·00	0·26	0·00
dates change	-0·05	NA	-0·08	NA
bwc20 change	0·45	NA	NA	NA
serial7 change	-0·04	NA	NA	NA
scissor change	0·41	NA	NA	NA
cactus change	0·25	NA	NA	NA
president change	-0·02	NA	NA	NA
immediate recallchange	-0·06	NA	-0·06	NA
delayed recallchange	-0·10		-0·12	NA
dates missing	0·00	0·00	0·00	0·00
bwc20 missing	0·00		NA	NA
serial7 missing	0·00	0·00	NA	NA
scissor missing	0·00	NA	NA	NA
cactus missing	0·00	NA	NA	NA
president missing	0·00	0·00	NA	NA
immediate recallmissing	0·00	0·00	0·00	0·00
delayed recallmissing	0·00	0·00	0·00	0·00
ADL missing	0·00	0·00	0·00	0·00
IADL missing	0·00	0·00	0·00	0·00
dates changemissing	-0·09	NA	-0·09	NA
bwc20 changemissing	0·00	NA	NA	NA
serial7 changemissing	0·00	NA	NA	NA
scissor change missing	0·00	NA	NA	NA
cactus changemissing	0·00	NA	NA	NA
president changemissing	0·00	NA	NA	NA
immediate recall change missing	0·00	NA	0·00	NA
delayed recall change missing	0·00	NA	0·00	NA
ADLchange missing	1·15	4·17	1·24	3·28
IADLchange missing	-0·31	-4·45	-0·44	-4·04
IQCODE	NA	-1·79	NA	NA
proxy	NA	0·00	NA	NA
IQCODE change	NA	1·11	NA	NA
IQCODE missing	NA	-5·47	NA	NA

© 2025 Dou X et al. *JAMA Network Open.*

proxy missing	NA	0·00	NA	NA
IQCODE changemissing	NA	0·19	NA	NA
/cut1	0·18	7·30	0·98	-1·43
/cut2	1·66	-5·72	2·41	-0·13

Note: *'Change'* represents the difference in cognitive function scores from the TICS assessment between the prior wave and the current wave. *'Missing'* indicates whether cognitive function variables are missing. Missing values are coded as 1 if an observation is missing. *'Change Missing'* refers to cases where the variables for "change" are missing. *'Cut1'* and *'cut2'* are the cut points that define the boundaries between ordinal categories for dementia status. If the latent variable falls between cut1 and cut2, the individual is classified as living withdementia.

© 2025 Dou X et al. *JAMA Network Open.*

eTable 7. Estimated Prevalence of Dementia in the United States, Europe, England

	the US	Europe	England
1890-1913	41.24%	NA	NA
1914-1918 / 1901-1918 / 1908-1918	22·54%	44·56%	28·99%
1919-1923	15·17%	34·07%	18·76%
1924-1928	12·31%	25·72%	13·00%
1929-1933	9·77%	18·64%	8·52%
1934-1938	7·86%	12·31%	7·79%
1939-1943	4·14%	5·38%	8·16%
1944-1948	2·73%	3·38%	7·41%
Total	14·16%	14·58%	11·00%
Number of observations	99 420	83 580	31 384

© 2025 Dou X et al. *JAMA Network Open.*

eTable 8. Estimated Prevalence of Dementia for Females and Males in the US, Europe and England

	Female sample			Male sample		
	the US	Europe	England	the US	Europe	England
1890-1913	46.61%	NA	NA	30.25%	NA	NA
1914-1918 /1901-1918 /1908-1918	26·17%	49·28%	3·37%	16·16%	33·84%	21·10%
1919-1923	17·35%	38·46%	21·03%	11·88%	26·89%	14·95%
1924-1928	13·71%	30·24%	14·97%	10·54%	19·50%	10·42%
1929-1933	10·87%	21·47%	10·36%	8·24%	15·16%	6·36%
1934-1938	8·54%	14·98%	7·855%	6·97%	9·33%	7·71%
1939-1943	4·55%	6·20%	7·67%	3·59%	4·48%	8·71%
1944-1948	2·66%	3·61%	6·28%	2·85%	3·13%	8·67%
Total	16·55%	17·55%	12·44%	10·69%	10·99%	9·19%
Number of observations	58 883	45 722	17 502	40 537	37 858	13 882

© 2025 Dou X et al. *JAMA Network Open.*

eTable 9. Cohort Effects Estimated for Females in the US, Europe and England

	the US	Europe	England
1919-1923	-0·205***	-0·228***	-0·310***
	(-0·300 to -0·111)	(-0·365 to -0·091)	(-0·504 to -0·116)
1924-1928	-0·313***	-0·417***	-0·427***
	(-0·432 to -0·194)	(-0·571 to -0·263)	(-0.678 to -0.176)
1929-1933	-0·346***	-0·589***	-0·562***
	(-0·495 to -0·197)	(-0·771 to -0·407)	(-0.867 to -0.258)
1934-1938	-0·317***	-0·780***	-0·721***
	(-0·496 to -0·138)	(-1·001 to -0·560)	(-1.104 to -0.338)
1939-1943	-0·449***	-1·180***	-0·691***
	(-0·663 to -0·234)	(-1·435 to -0·925)	(-1·152 to -0·231)
1944-1948	-0·580***	-1·504***	-0·763***
	(-0·857 to -0·303)	(-1·795 to -1·213)	(-1·296 to -0·230)
Number of observations	58 883	45 722	17 502

Note: In all regressions we control for age groups and GDP growth rate for each region. 95% confidence intervals in parentheses; * $p < 0{\cdot}10$, ** $p < 0{\cdot}05$, *** $p < 0{\cdot}01$

eTable 10. Cohort Effects Estimated for Males in the US, Europe and England

	the US	Europe	England
1919-1923	-0·104*	-0·190**	-0·066
	(-0·227 to 0·020)	(-0·376 to -0·005)	(-0·331 to 0·198)
1924-1928	-0·161**	-0·419***	-0·146
	(-0·317 to 0·006)	(-0·621 to -0·217)	(-0.456 to 0.164)
1929-1933	-0·251**	-0·536***	-0·319
	(-0·445 to -0·057)	(-0·769 to -0·302)	(-0.694 to 0.056)
1934-1938	-0·257**	-0·792***	-0·173
	(-0·493 to -0·020)	(-1·063 to -0·520)	(-0.652 to 0.305)
1939-1943	-0·454***	-1·061***	-0·064
	(-0·738 to -0·170)	(-1·373 to -0.750)	(-0·606 to 0·477)
1944-1948	-0·483***	-1·341***	-0·074
	(-0·835 to -0·132)	(-1·696 to -0·986)	(-0·689 to 0·541)
Number of observations	40 537	37 858	13 882

Note: In all regressions we control for age groups and GDP growth rate for each region. 95% confidence intervals in parentheses; * $p < 0{\cdot}10$, ** $p < 0{\cdot}05$, *** $p < 0{\cdot}01$

© 2025 Dou X et al. *JAMA Network Open.*

eTable 11. Cohort Effects Estimated in the US, Europe and England

	the US	Europe	England
1919-1923	-0·092***	-0·309***	-0·163**
	(-0·159 to -0·024)	(-0·420 to -0·198)	(-0·316 to -0·010)
1924-1928	-0·154***	-0·550***	-0·253***
	(-0·217 to -0·091)	(-0·674 to -0·427)	(-0.443 to -0.063)
1929-1933	-0·213***	-0·705***	-0·397***
	(-0·278 to -0·147)	(-0·849 to -0·560)	(-0.629 to -0.165)
1934-1938	-0·221***	-0·906***	-0·423***
	(-0·293 to -0·150)	(-1·077 to -0·736)	(-0.721 to -0.125)
1939-1943	-0·150***	-1·201***	-0·392**
	(-0·229 to -0·072)	(-1·396 to -1·007)	(-0·743 to -0·041)
1944-1948	-0·237***	-1·440***	-0·500**
	(-0·331 to -0·142)	(-1·666 to -1·215)	(-0·902 to -0·098)
Number of observations	99 420	83 580	31 384

Note: In all regressions we control for age groups and survey year for each region. 95% confidence intervals in parentheses; * p < 0·10, ** p < 0·05, *** p < 0·01

© 2025 Dou X et al. *JAMA Network Open.*

eTable 12. Cohort Effects Estimated for Females in the US, Europe and England

	the US	Europe	England
1919-1923	-0·096**	-0·292***	-0·238**
	(-0·179 to -0·012)	(-0·431 to -0·153)	(-0·427 to -0·049)
1924-1928	-0·180***	-0·508***	-0·336***
	(-0·261 to -0·100)	(-0·664 to -0·353)	(-0.581 to -0.091)
1929-1933	-0·250***	-0·674***	-0·465***
	(-0·334 to -0·165)	(-0·858 to -0·490)	(-0.763 to -0.167)
1934-1938	-0·237***	-0·842***	-0·621***
	(-0·330 to -0·145)	(-1·062 to -0·623)	(-1.002 to -0.240)
1939-1943	-0·152***	-1·179***	-0·640**
	(-0·255 to -0·050)	(-1·430 to -0·928)	(-1·101 to -0·179)
1944-1948	-0·219***	-1·435***	-0·811**
	(-0·341 to -0·096)	(-1·726 to -1·143)	(-1·342 to -0·281)
Number of observations	58 883	45 722	17 502

Note: In all regressions we control for age groups and survey year for each region. 95% confidence intervals in parentheses; * *p* < 0·10, ** *p* < 0·05,*** *p* < 0·01

© 2025 Dou X et al. *JAMA Network Open.*

eTable 13. Cohort Effects Estimated for Males in the US, Europe and England

	the US	Europe	England
1919-1923	-0·084	-0·279***	-0·025
	(-0·199 to 0·031)	(-0·467 to -0·090)	(-0·283 to 0·233)
1924-1928	-0·089*	-0·536***	-0·090
	(-0·192 to 0·013)	(-0·740 to -0·332)	(-0.392 to 0.213)
1929-1933	-0·121**	-0·646***	-0·253
	(-0·225 to -0·016)	(-0·882 to -0·411)	(-0.620 to 0.114)
1934-1938	-0·175***	-0·881***	-0·103
	(-0·288 to -0·061)	(-1·152 to -0·610)	(-0.576 to 0.370)
1939-1943	-0·124**	-1·110***	-0·013
	(-0·248 to -0·001)	(-1·419 to -0·800)	(-0·553 to 0·528)
1944-1948	-0·248***	-1·324***	-0·054
	(-0·397 to -0·099)	(-1·682 to -0·966)	(-0·670 to 0·561)
Number of observations	40 537	37 858	13 882

Note: In all regressions we control for age groups and survey year for each region. 95% confidence intervals in parentheses; * p < 0·10, ** p < 0·05,*** p < 0·01

© 2025 Dou X et al. *JAMA Network Open.*

eTable 14. Robustness Check Results of Age, Cohort, Period for Individuals in the US, Europe and England

	the US	Europe	England
1919-1923	-0·76[c]	-0·39[c]	-0·34[c]
	(-0·84 to -0·68)	(-0·51 to -0·27)	(-0·49 to -0·19)
1924-1928	-0·87[c]	-0·64[c]	-0·47[c]
	(-0·97 to -0·76)	(-0·77 to -0·52)	(-0·66 to -0·28)
1929-1933	-0·96[c]	-0·91[c]	-0·67[c]
	(-1·09 to -0·83)	(-1·06 to -0·76)	(-0·90 to -0·45)
1934-1938	-1·077[c]	-1·10[c]	-0·18[c]
	(-1·23 to -0·92)	(-1·27 to -0·93)	(-1·0 to -0·43)
1939-1943	-1·24[c]	-1·31[c]	-0·73[c]
	(-1·43 to -1·05)	(-1·50 to -1·12)	(-1·06 to -0·39)
1944-1948	-1·32[c]	-1·76[c]	-0·81[c]
	(-1·55 to -1·09)	(-1·97 to -1·54)	(-1·20 to -0·42)
Number of observations	99 420	83 580	31 384

Note: In all regressions we control for age groups and GDP growth rate for each region. 95% confidence intervals in parentheses; $p < 0·10$, $p < 0·05$ [b], $p < 0·01$[c]

© 2025 Dou X et al. *JAMA Network Open.*

eTable 15. Robustness Check Results of Age, Cohort, Period for Females in the US, Europe and England

	the US	Europe	England
1919-1923	-1·023***	-0·385***	-0·439***
	(-1·126to-0·919)	(-0·538to-0·233)	(-0·629to-0·250)
1924-1928	-1·183***	-0·646***	-0·635***
	(-1·316to-1·050)	(-0·811to-0·481)	(-0·878to-0·393)
1929-1933	-1·324***	-0·952***	-0·843***
	(-1·489to-1·159)	(-1·143to-0·761)	(-1·133to-0·554)
1934-1938	-1·481***	-1·084***	-0·959***
	(-1·678to-1·284)	(-1.307to-0·860)	(-1·330to-0·588)
1939-1943	-1·658***	-1·334***	-0·961***
	(-1·892to-1·424)	(-1·587to-1·081)	(-1·403to-0·519)
1944-1948	-1·849***	-1·678***	-1·032***
	(-2·138to-1·560)	(-1·964to-1·392)	(-1·543to-0·520)
Number of observations	58 883	45 722	17 502

Note: In all regressions we control for age groups and GDP growth rate for each region. 95% confidence intervals in parentheses; * p< 0·10, ** p< 0·05, *** p<0·01

© 2025 Dou X et al. *JAMA Network Open.*

eTable 16. Robustness Check Results of Age, Cohort, Period for Males in the US, Europe and England

	the US	Europe	England
1919-1923	-0·227***	-0·306***	-0·156
	(-0·353 to -0·101)	(-0·502 to -0·110)	(-0·406 to 0·095)
1924-1928	-0·197***	-0·520***	-0·134
	(-0·358 to -0·036)	(-0·728 to -0·311)	(-0·432 to 0·164)
1929-1933	-0·202***	-0·691***	-0·321
	(-0·405 to -0·002)	(-0·931 to- 0·451)	(-0·679 to 0·037)
1934-1938	-0·212***	-0·956***	-0·263*
	(-0·458 to -0·034)	(-1·230 to -0·681)	(-0·720 to 0·193)
1939-1943	-0·340***	-1·096***	-0·284
	(-0·634 to -0·045)	(-1·403 to -0·790)	(-0·806 to 0·237)
1944-1948	-0·196***	-1·698***	-0·389
	(-0·558 to -0·166)	(-2·043 to -1·353)	(-0·987 to 0·210)
Number of observations	40 537	37 858	13 882

Note: In all regressions we control for age groups and GDP growth rate for each region. 95% confidence intervals in parentheses; * p< 0·10, ** p< 0·05, *** p<0·01

© 2025 Dou X et al. *JAMA Network Open.*

eTable 17. Summary of Missing Data for Key Variables

	the US	Europe	England
dates missing	12 362 (12.43%)	344 (0.4%)	2014 (6.42%)
bwc20 missing	11 222 (11.29%)	NA	NA
serial7 missing	11 222 (11.29%)	NA	NA
scissor missing	12 362 (12.43%)	NA	NA
cactus missing	12 362 (12.43%)	NA	NA
president missing	12 362 (12.43%)	NA	NA
immediate recall missing	23 240 (23.38%)	4 121 (4.93%)	1871 (5.96%)
delayed recall missing	17 417 (17.52%)	4 064 (4.86%)	1797 (5.73%)
ADL missing	140 (0.14%)	427 (0.51%)	101 (0.32%)
IADL missing	246 (0.25%)	427 (0.51%)	101 (0.32%)
Number of observations	99 420	83 580	31 384

© 2025 Dou X et al. *JAMA Network Open.*

eReference.

[1] Kingma DP, Ba J. Adam: A Method for Stochastic Optimization. arXiv; 2017.

© 2025 Dou X et al. *JAMA Network Open.*
