# Supplementary material for: Generational Differences in Age-Specific Dementia Prevalence Rates
Source: JAMA Netw Open. 2025 Jun 2;8(6):e2513384. doi: 10.1001/jamanetworkopen.2025.13384 (PMC12551361; doi:10.1001/jamanetworkopen.2025.13384)
Supplement: Supplement 2. — Data Sharing Statement [file jamanetwopen-e2513384-s002.pdf]

Data Sharing Statement

Dou. Generational Differences in Age-Specific Dementia Prevalence Rates. *JAMA Netw Open*. Published June 02, 2025. doi:10.1001/jamanetworkopen.2025.13384

Data
Data available: No
